# Supplementary material for: The transcription factor EPAS1 links DOCK8 deficiency to atopic skin inflammation via IL-31 induction
Source: Nat Commun. 2017 Jan 9;8:13946. doi: 10.1038/ncomms13946 (PMC5228069; doi:10.1038/ncomms13946)
Supplement: Supplementary Information — Supplementary Figures and Supplementary Tables [file ncomms13946-s1.pdf]

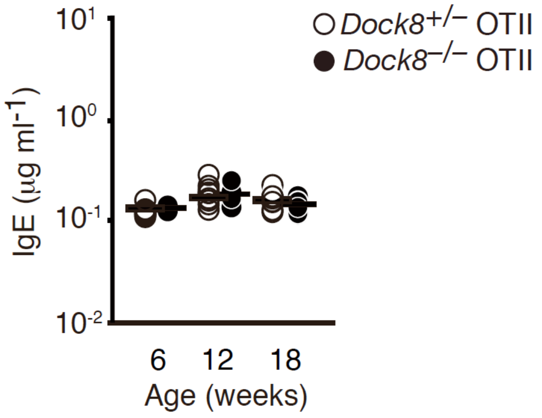

Supplementary Figure 1 | Comparison of serum IgE levels between *Dock8*$^{+/–}$ and *Dock8*$^{–/–}$ OTII Tg mice. Serum concentrations of IgE in *Dock8*$^{+/–}$ and *Dock8*$^{–/–}$ OTII Tg mice were compared at 6 (*n* = 10), 12 (*n* = 9) and 18 (*n* = 9) weeks old. The lines indicate the mean values.

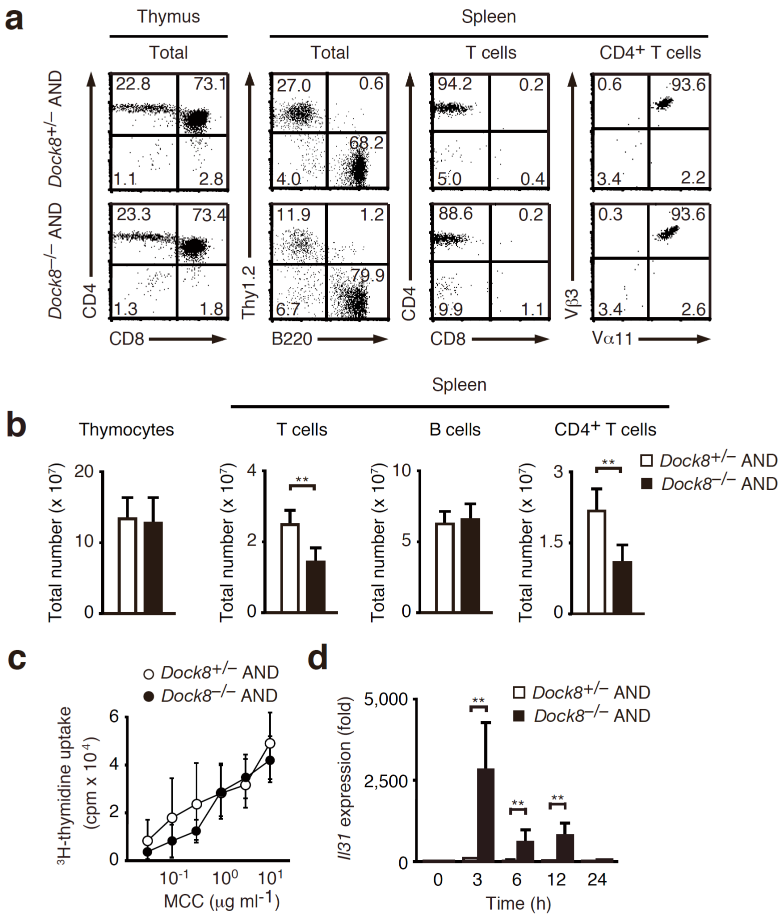

Supplementary Figure 2 | DOCK8 acts as a negative regulator for IL-31 induction in *Dock8*$^{-/-}$ AND Tg mice. (**a**,**b**) Flow cytometric analyses of thymocytes and spleen cells from 6–8-week-old *Dock8*$^{+/-}$ and *Dock8*$^{-/-}$ AND Tg mice. The numerals in quadrants indicate the percentage of each subset of leukocytes. Data are expressed as mean ± s.d. of 5 mice per group. $^{**}P < 0.01$ (two-tailed Student's *t*-test). See Supplementary Fig. 10 for FACS gating strategy. (**c**) Antigen-specific proliferation of CD4^{+} T cells from *Dock8*$^{+/-}$ and *Dock8*$^{-/-}$ AND Tg mice. Data are expressed as mean ± s.d. of 9 samples per group. (**d**) *Il31* gene expression in CD4^{+} T cells from *Dock8*$^{+/-}$ and *Dock8*$^{-/-}$ AND Tg mice after secondary stimulation with anti-CD3ε and anti-CD28 antibodies. Expression (fold increase) is relative to that of *Dock8*$^{+/-}$ samples without secondary stimulation. Data are expressed as mean ± s.d. of 7 samples per group. Comparison was made between two groups at the same time point. $^{**}P < 0.01$ (two-tailed Student's *t*-test).

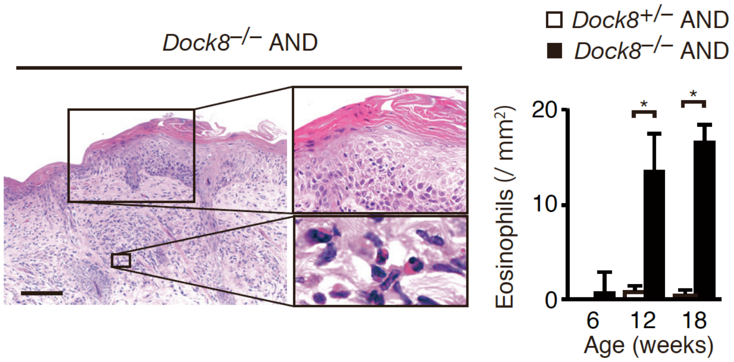

Supplementary Figure 3 | Haematoxylin and eosin staining of the skin from 18-week-old *Dock8*$^{-/-}$ AND Tg mice. Scale bar, 100 μm. Magnified views of the boxed area show spongiosis (top) and the presence of eosinophils (bottom). The number of eosinophils per mm^2 was compared between *Dock8*$^{+/-}$ and *Dock8*$^{-/-}$ AND Tg mice. Data are expressed as mean ± s.d. of 4 mice group. $^*P < 0.05$ (two-tailed Mann-Whitney test).

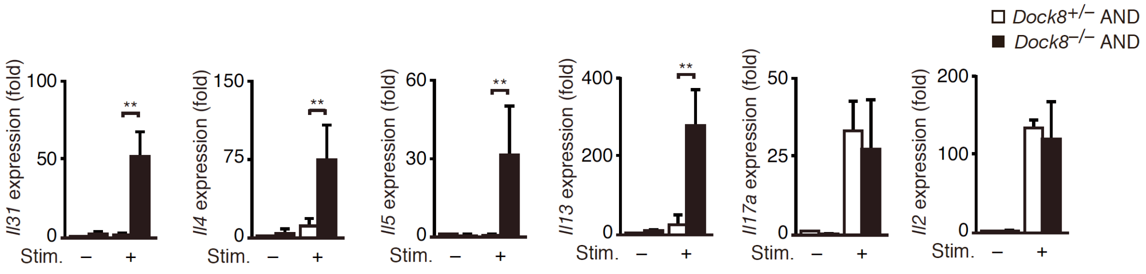

Supplementary Figure 4 | The skin-infiltrating CD4+ T cells in *Dock8*–/– AND Tg mice express Th2 cytokines. The expression of cytokine genes in dermal CD4+ T cells upon TCR stimulation. Expression (fold increase) is relative to that of the unstimulated samples. Data are expressed as mean ± s.d. of 6 samples per group. $**P < 0.01$ (two-tailed Student's *t*-test).

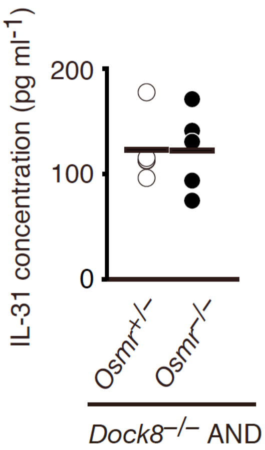

Supplementary Figure 5 | Comparison of serum IL-31 levels between *OSMR*$^{+/–}$ and *OSMR*$^{–/–}$ *Dock8*$^{–/–}$ AND Tg mice. Serum concentrations of IL-31 in *OSMR*$^{+/–}$ and *OSMR*$^{–/–}$ *Dock8*$^{–/–}$ AND Tg mice were compared at 18 weeks old (n = 5). The lines indicate the mean values.

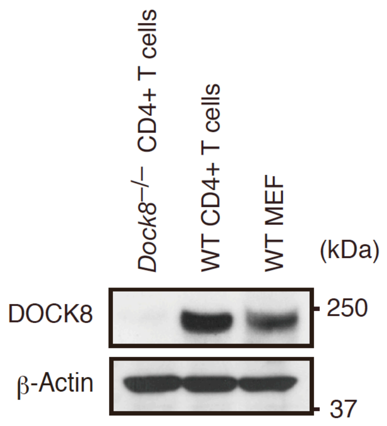

Supplementary Figure 6 | Expression of DOCK8 in MEFs. Cell extracts were prepared from $CD4^+$ T cells (WT and *Dock8*$^{-/-}$ mice) and WT MEFs, and were subjected to Western blot analysis using anti-DOCK8 and anti-βactin antibodies.

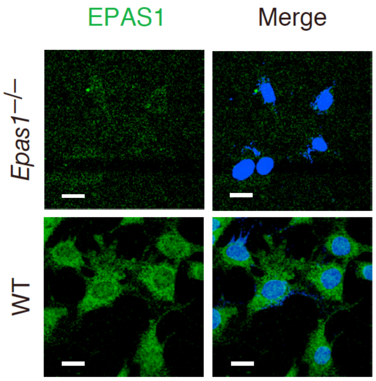

Supplementary Figure 7 | Validation of anti-EPAS1 antibody used in immunofluorescence analyses. Staining profiles of WT and *Epas1*$^{-/-}$ MEFs are shown. DAPI was used to stain nuclei. Scale bars, 20 μm.

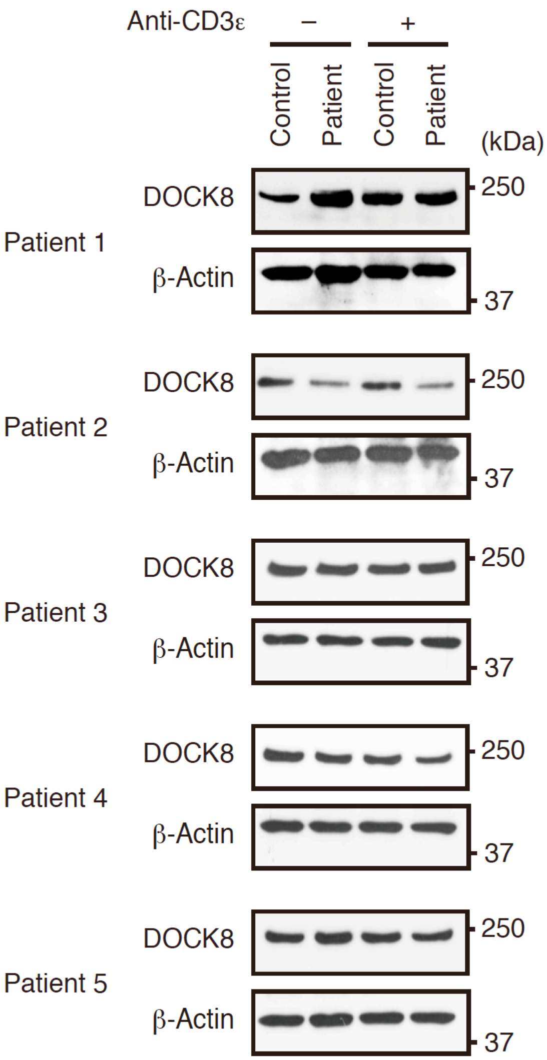

Supplementary Figure 8 | Comparable expression of DOCK8 in $CD4^+$ T cells from AD patients and healthy controls. Following cultivation of $CD4^+$ T cells in a 24-well plate coated with or without anti-CD3ε antibody for 6 h, DOCK8 expression in $CD4^+$ T cells was analysed by immunoblotting.

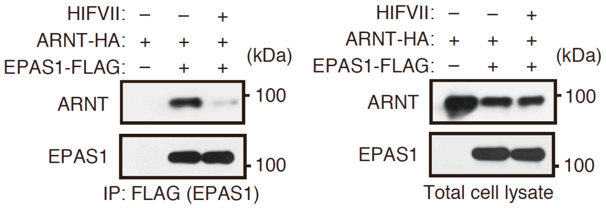

Supplementary Figure 9 | Effect of HIFVII on association between EPAS1 and ARNT. Following expression of FLAG-tagged EPAS1 and HA-tagged ARNT in HEK-293T cells, association between EPAS1 and ARNT was analysed in the presence or absence of HIFVII (30 μM). Data are representative of three independent experiments.

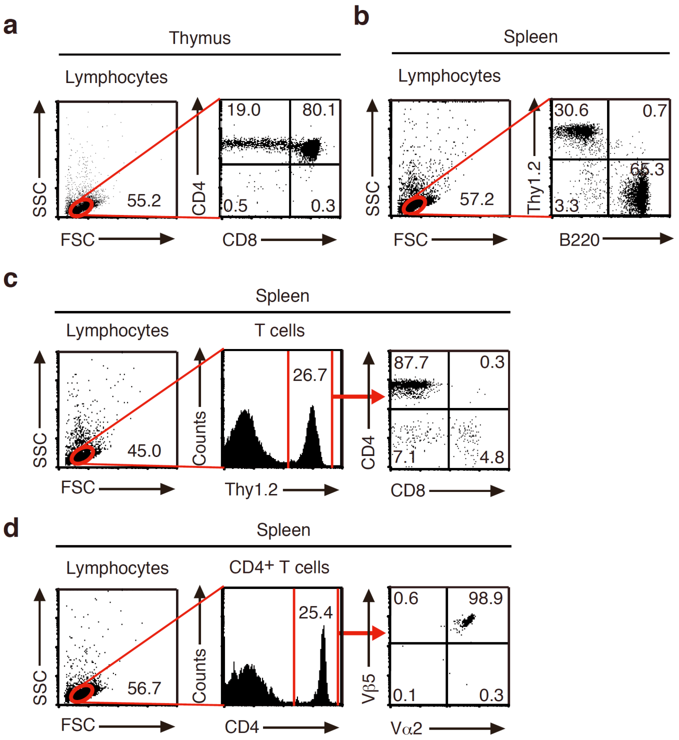

Supplementary Figure 10 | FACS gating strategy used in Figure 1a. (**a**,**b**) The gating strategy used to analyse the cell-surface expression of CD4, CD8, Thy1.2 (CD90.2) and/or B220 (CD45R) on total thymocytes and spleen cells. (**c**) The gating strategy used to analyse the cell-surface expression of CD4 and CD8 on Thy1.2^{+} splenic T cells. (**d**) The gating strategy used to analyse the cell-surface expression of Vα2 and Vβ5 on splenic CD4^{+} T cells.

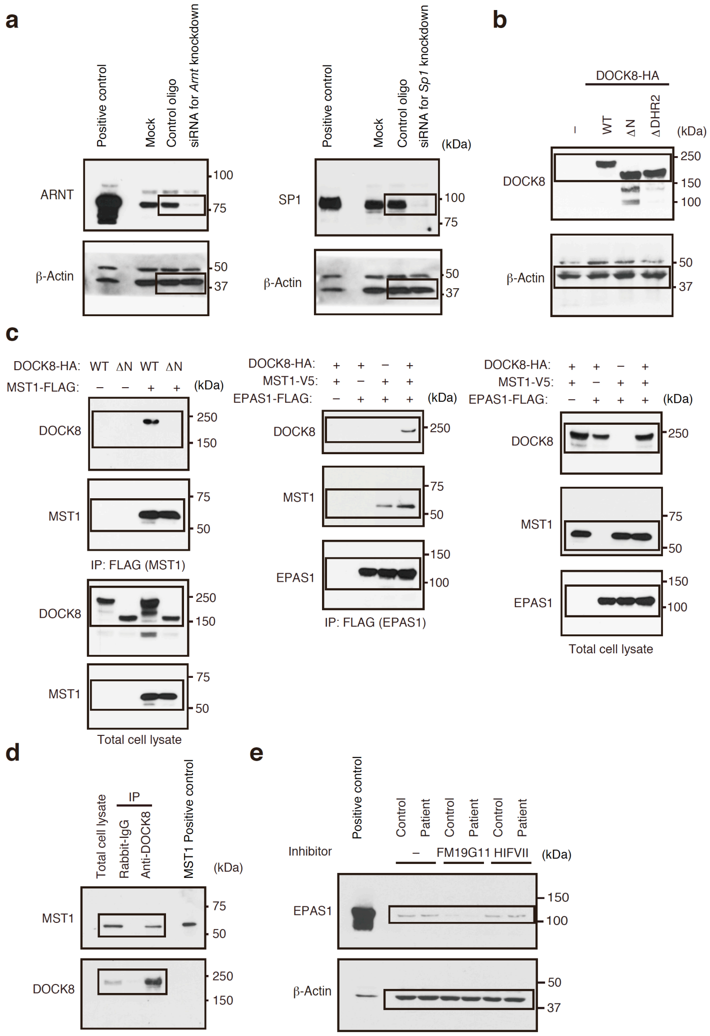

Supplementary Figure 11 | Uncropped scans of the Western blots. (**a**) Uncropped scans of the Western blots shown in Figure 5b. Positive control indicates HEK-293T cell extracts expressing pcDNA-ARNT-HA (left) or pcDNA-SP1-V5 (right). Mock, untreated MEF lysates. (**b**) Uncropped scans of the Western blots shown in Figure 6b. (**c**) Uncropped scans of the Western blots shown in Figure 6d. (**d**) Uncropped scans of the Western blots shown in Figure 6e. Positive control indicates HEK-293T cell extracts expressing pcDNA-MST1-V5. (**e**) Uncropped scans of the Western blots shown in Figure 7e. Positive control indicates HEK-293T cell extracts expressing pcDNA-EPAS1-FLAG.

Supplementary Table 1 │ Transcription factors differently expressed in activated CD4^{+} T cells from *Dock8*$^{+/–}$ and *Dock8*$^{–/–}$ AND Tg mice.

List number	Gene name	Z-score	Z-ratio*	*Dock8*$^{+/–}$ AND Tg Signal	*Dock8*$^{–/–}$ AND Tg Signal
1	*Batf2*	6.054390159	3.615418102	1421.913	5140.81
2	*Mamstr*	5.114130366	24.37466596	4.394995	107.126535
3	*Tbx21*	4.750302424	2.741328011	2987.6795	8190.2095
4	*Batf3*	4.309980069	2.496758712	2801.8955	6995.657
5	*Ajuba*	4.246428907	2.960550293	274.272	811.99605
6	*Sox12*	4.189911176	13.68761314	9.8169015	134.36995
7	*Epas1*	4.178403297	2.428002711	824.1352	2001.0025
8	*Gfi1*	3.772857413	1.979372381	5880.417	11639.535
9	*Esr1*	3.743103396	10.35550974	7.468642	77.341595
10	*Atf3*	3.632725431	2.162490159	3184.8975	6887.3095
11	*Klf17*	3.535121188	9.094421931	7.8169075	71.090255
12	*Maf*	3.521061799	2.459331282	519.41335	1277.4095
13	*Jdp2*	3.080611771	2.197369027	779.1263	1712.028
14	*Klf4*	3.065655669	1.917288313	924.21885	1771.994
15	*Ifi204*	2.895831887	1.68860953	5904.19	9969.8715
16	*Etv6*	2.671272723	1.763344721	2751.8465	4852.454
17	*Stat2*	2.623324545	1.745491939	3371.5435	5885.002
18	*Fosl1*	2.581422651	2.448300343	80.351355	196.72425
19	*Cited1*	2.578193795	5.003743099	32.33951	161.8186
20	*Jun*	2.544284329	1.584425847	7257.5375	11499.03
21	*Hif1a*	2.538211437	1.582683758	5029.313	7959.812
22	*Nfil3*	2.506781342	1.573698186	6755.2375	10630.705
23	*Hmox1*	2.411310819	1.546715677	10014.2775	15489.24
24	*Cebpe*	2.406260935	1.666906096	1086.6125	1811.281
25	*Fosl2*	2.388469328	1.660623906	1069.103	1775.378
26	*Rora*	2.372133869	1.833256421	487.3513	893.4399
27	*Irf7*	2.362097484	1.828557525	637.63895	1165.9595
28	*Pou4f1*	2.329520671	2.244227222	69.325155	155.5814
29	*Etv5*	2.317445706	1.807797737	654.55525	1183.3035
30	*Trp73*	2.296821033	4.197571138	9.113487	38.25451
31	*Gata2*	2.286631205	2.211216496	84.760425	187.42365
32	*Id2*	2.284644354	1.511629124	5315.74139	8035.4295
33	*L3mbtl4*	2.230225584	21.87445325	3.497932	76.51535
34	*Vdr*	2.200379051	1.754482493	405.45235	711.35905
35	*Egr2*	2.152819179	1.579615749	2410.302	3807.351
36	*Irf5*	2.141055969	1.575677169	3423.3145	5394.0385
37	*Tnfrsf8*	2.126463292	3.77401759	24.47883	92.383535
38	*Ebf1*	2.109422355	2.079893488	90.175435	187.5553
39	*Tead2*	2.109112214	3.733352586	15.997455	59.72414
40	*Tcf4*	2.039810673	1.542181476	1192.9585	1839.7585

*Z-ratio was calculated by dividing the values of *Dock8*$^{–/–}$ AND Tg samples by those of *Dock8*$^{+/–}$ AND Tg samples.

Supplementary Table 2 | Demographic information for AD patients and healthy controls.

AD<br>n=6	Age (year)<br>Mean ± median<br>35.2 ± 4.7	Sex	Severity
Patient 1	33	Male	moderate
Patient 2	30	Male	moderate
Patient 3	43	Male	severe
Patient 4	29	Female	moderate
Patient 5	54	Female	moderate
Patient 6	22	Male	moderate

Healthy Controls<br>n=6	Age (year)<br>Mean ± median<br>29.0 ± 1.3	Sex
Control 1	24	Male
Control 2	33	Male
Control 3	28	Male
Control 4	31	Female
Control 5	28	Female
Control 6	30	Female

Supplementary Table 3 | The primers used for conventional RT-PCR and real-time PCR in this study.

Conventional RT-PCR

Gapdh	5′-CAGTATGACTCCACTCACGGC-3′<br>5′-TCACGCCACAGCTTTCCAGAG-3′
Il31	5′-AAACAAGAGTCTCAGGATCTTTATAACAAC-3′<br>5′-AGCCATCTTATCACCCAAGAA-3′
Epas1	5′-GAGCAAAGGGTCCCTGTCTC-3′<br>5′-AATTCATCGGGGGCCATGTT-3′
Mst1	5′-GGCAGCGTGTACAAGGCTAT-3′<br>5′-ATATGGGGGCTTTCCTTCAG-3′
GAPDH	5′-CCCATCACCATCTTCCAG-3′<br>5′-ATGACCTTGCCCACAGCC-3′
DOCK8	5′-CGACTTGGACGTGGTGTTCACG-3′<br>5′-TTCTGTTGCCCAGGTGTTCCTTG-3′
EPAS1	5′-GAGTCCTCAGCCTATGGCAAG-3′<br>5′-CGGCATCAAAGAAGATGGAG-3′

Real-time PCR

Hprt	5′-CTGGTGAAAAGGACCTCTCG-3′<br>5′-TGAAGTACTCATTATAGTCAAGGGCA-3′
Il31	5′-AAACAAGAGTCTCAGGATCTTTATAACAAC-3′<br>5′-ACGGCAGCTGTATTGATTCGT-3′
Il4	5′-CGAAGAACACCACAGAGAGTGAGCT-3′<br>5′-GACTCATTCATGGTGCAGCTTATCG-3′
Il5	5′-AAAGAGAAGTGTGGCGAGGAGA-3′<br>5′-CACCAAGGAACTCTTGCAGGTAA-3′
Il13	5′-GAGCAACATCACACAAGACCAGA-3′<br>5′-GGCCAGGTCCACACTCCATA-3′
Il17a	5′-CTCCAGAAGGCCCTCAGACTAC-3′<br>5′-GGGTCTTCATTGCGGTGG-3′
Il2	5′-CCTGAGCAGGATGGAGAATTACA-3′<br>5′-TCCAGAACATGCCGCAGAG-3′
GAPDH	5′-GTGAAGGTCGGAGTCAACG-3′<br>5′-GGTGAAGACGCCAGTGGACTC-3′
IL31	5′-TGGACCTCGCACTAAAATCATTG-3′<br>5′-CGAAAGGAAGAGATGGCCTTAA-3′
IL2	5′-GCAACTCCTGTCTTGCATTG-3′<br>5′-CAGTTCTGTGGCCTTCTTGG-3′
